# P027. Idiopathic intracranial hypertension without papilledema in refractory chronic daily headache

**DOI:** 10.1186/1129-2377-16-S1-A108

**Published:** 2015-09-28

**Authors:** Valentina Favoni, Francesco Toni, Sabina Cevoli, Luigi Cirillo, Chiara La Morgia, Giulia Giannini, Rossana Terlizzi, Hana Privitera Hrustemovic, Monica Messia, Pietro Cortelli, Giulia Pierangeli

**Affiliations:** Department of Biomedical and NeuroMotor Sciences (DIBINEM) Alma Mater Studiorum-University of Bologna, IRCCS Institute of Neurological Sciences of Bologna, Bologna, Italy; Neuroradiology Department, IRCCS Institute of Neurological Sciences of Bologna, Bologna, Italy

## Background

A diagnosis of idiopathic intracranial hypertension without papilledema (IIHWOP) should be considered in unresponsive chronic daily headache (CDH) patients[1]. A CSF opening pressure (OP) above 200 mm H2O has been detected in chronic migraine patients with conflicting result, ranging from 10% to 86% of patients[1, 2]. Moreover, controversies exist regarding the OP cut-off value greater than 200 or 250 mm H2O and the role of transverse sinus stenosis (TSS)[3, 4].

## Aim

To investigate the frequency of IIHWOP and TSS in adult patients with refractory CDH.

## Methods

In a prospective study, patients with refractory CDH underwent ophthalmologic evaluation and Optical Coherence Tomography to rule out the presence of papilledema; cerebral MR venography (MRV) to detect TSS; and a lumbar puncture to measure OP. In patients showing an OP < 200 mmH2O the procedure was stopped after a 6 mL CSF withdrawal for routine analysis. In subjects with an OP > 200 mm H2O, intracranial pressure measurements were repeated every 2 mL of extracted CSF, up to about 100 mm H2O. An MRV was repeated 1 month after LP in patients with OP > 200 mmH2O. TSS was identified using a combined conduit score (CCS).

## Results

Thirty-six patients were enrolled. Five patients were excluded due to protocol violations. Analyses were conducted in 31 patients (24 F, 7 M; mean age 50.4±11; mean BMI 26.5±6.5). None of the patients had papilledema. All displayed an OP lower than 250 mm H2O (range 102-245). Six patients (19%) had an OP greater than 200 mm H2O: three of them achieved an improvement of headache frequency or intensity after 8-18 ml CSF withdrawal. Fifteen patients (48%) had MRV evidence of TSS: bilateral in 4 and unilateral in 11. Using a Pearson's correlation coefficient test, no significant correlation between CCS and OP was found. After CSF withdrawal, no changes of CCS were found in the six patients who repeated MRV.

## Conclusions

In our series, all patients displayed normal OP values (< 250 mm H2O). Nineteen percent of patients had an OP greater than 200 mm H2O. Our results confirm a low prevalence of IIHWOP in chronic headache sufferers. Moreover, the prevalence of sinus venous stenosis (50%) was lower than previously described in unresponsive chronic headache patients (92.8%), but similar to a series of unselected chronic headache patients (50.6%)[1, 5]. Transverse sinus stenosis seems not to correlate with CSF opening pressure, putting its role into question.

Written informed consent to publication was obtained from the patient(s).
